# First Feeding of Cuttlefish Hatchlings: Pioneering Attempts in Captive Breeding

**DOI:** 10.3390/ani14131993

**Published:** 2024-07-06

**Authors:** Francesca Maradonna, Andrea Pessina, Ghasem Ashouri, Emilio Notti, Giulia Chemello, Giulia Russo, Giorgia Gioacchini, Oliana Carnevali

**Affiliations:** 1 Department of Life and Environmental Sciences, Polytechnic University of Marche, Via Brecce Bianche, 60131 Ancona, Italy; f.maradonna@staff.univpm.it (F.M.); a.pessina88@gmail.com (A.P.); ghasem.ashouri@yahoo.com (G.A.); g.chemello@staff.univpm.it (G.C.); giu-rus@hotmail.com (G.R.); giorgia.gioacchini@staff.univpm.it (G.G.); 2 Italian National Research Council (CNR), Institute of Marine Biological Resources and Biotechnologies (IRBIM), Largo Fiera della Pesca 1, 60125 Ancona, Italy; emilio.notti@cnr.it

**Keywords:** cuttlefish, aquaculture, hatchlings, health status, Adriatic Sea

## Abstract

**Simple Summary:**

In this study, the first evidence regarding the possibility of breeding cuttlefish in captivity using dry pellet feed is provided. These results are extremely important because they can meet the increasing demand of the market and thus could limit the reduction in natural stocks due to the growing fishing effort. The results obtained from the pilot study allowed the selection of a commercial pellet-based diet which ensured a similar growth rate and intestinal maturity, with respect to a diet based on frozen krill, which closely resembled the feeding habits of cuttlefish in their natural environment. Furthermore, the administration of this pellet diet does not induce stress in hatchlings/larvae nor induces the activation of antioxidant system genes. Overall, the results are very encouraging and suggest the possibility of undertaking cuttlefish breeding on a larger scale.

**Abstract:**

In the last few decades, the cuttlefish market has grown to approximately 14% of the world’s fisheries, and operators have begun to express concerns about the decline of this resource. In this context, the production of cuttlefish through aquaculture could offer a diversifying and valuable response to the increasing market demand and help alleviate the environmental pressure on this species. Therefore, the aim of this study is to identify a dry, cost-effective, and easy-to-administer diet that can successfully support the initial phases of cuttlefish growth and provide a similar performance to a krill-based diet, which closely mimics their natural diet. To achieve this objective, cuttlefish hatchlings were distributed among different experimental tanks, each receiving one of the five different diets (namely Diets A to E). Mortality and morphological parameters were monitored until day 10 post hatching, and the two most effective diets (Diets A and B) were chosen for further trials. The results indicated that Diet B had similar survival and growth rates to Diet A, which was based on frozen krill. Histological analysis revealed a comparable degree of gut maturity between the organisms fed the two diets. Likewise, levels of amylase and trypsin enzymes and *hsp70*, *cat*, and *sod* mRNA did not exhibit significant differences between the two groups. In conclusion, our findings provide preliminary evidence supporting the possibility of cultivating cuttlefish in captivity using a pelleted diet, representing a promising starting point for larger-scale breeding efforts.

## 1. Introduction

Since the beginning of the 1970s, global fish consumption has doubled, causing a significant increase in aquaculture product demand. This resulted from the combined effects of the increasing world population, the decrease in wild catches and the change in consumer preferences. This scenario also affected the cuttlefish market, and the demand in 2020 reached approximately 14% of the world’s fisheries [1,2]. In Italy, mainly on the Adriatic side, this species represented 50–80% of the total catches obtained from bottom trawling within the coastal strip [3]. The official data collected from ISTAT and from representative Adriatic fish markets indicate considerable fluctuations in the production of this species from one year to the next, highlighting the seasonal nature of the cuttlefish and its catches [4]. Nevertheless, in the last few years, operators started to complain about the decline of this resource and recognize the need to realize breeding systems and restocking actions, aware of the importance of implementing a sustainable withdrawal activity and practicing a more environmentally respectful fishing. The scenario is even more complex if the data regarding the decline of the natural stocks are matched to the low fecundity of the species. Studies on cuttlefish reproductive biology revealed that most females lay a small number of eggs (from 300 to a maximum of 3000) once in their lifetime and benthonic hatchings have reduced dispersion capacity [5]. In this context, cuttlefish production by aquaculture could be a diversifying and a valuable response to the increasing market demand and could contribute to reducing the environmental pressure on this species. The first attempt to breed and rear cuttlefish in captivity started in the early 1960s [6,7], and although various strategies have been carried out, some substantial difficulties still exist. Three main factors still delay the transition from pilot-scale to full-scale culture production: (i) the necessity of live prey during the first part of the lifecycle, (ii) the lack of an adequate artificial diet for all life stages, and (iii) the full control of reproduction in captivity [8]. Regarding the first aspect, several papers so far reported that cephalopods could be reared with both live and dead prey [9] and, during the first part of their life, mysids, among live preys, promoted a better growth [10], but their use was economically unsustainable. Thus, studies have suggested that caprellids and gammarids could represent a valuable, less expensive alternative to mysids [11,12] but their large-scale culture is not widespread, and therefore, the market demand still relies on environmental catches, with unaffordable costs. For this reason, gammarids are scarcely present among the ingredients of pelleted feed used in aquaculture, including those intended for ornamental species [13].

Cuttlefish, different from other invertebrates, have a highly evolved nervous system [14] and the first hours after hatching are very important and capable of influencing the specimen’s future eating habits. The visual experience overcomes the innate food knowledge and thus influences food imprinting. In this phase, if the young cuttlefish receives information on the abundance of a specific type of prey, its food preference will be directed towards the prey that has most stimulated its attention [15]. In addition, since cuttlefish juveniles have not yet developed the vertical brain lobe, predation depends on the ability to spot prey movement, and unlike adults, they do not feed on dead or immobile prey [15]. Thus, an issue with this species’ aquaculture practices is represented by the transition from live feed to frozen food, which mainly rely on the quality of supplied food [16,17]. A big effort should be carried out to find a valuable diet, able to stimulate the specimen’s attention and satisfy the nutritional requirements and one that is possibly not time and cost consuming for the farmer. In the last few years, the need to find a balanced and easy-to-administer diet is gaining growing interest as result of the increased awareness among fishermen and public bodies regarding the recovery of eggs laid on traps and ropes, which would otherwise commonly be lost at the end of the fishing season. These eggs are commonly subjected to destructive actions during cleaning or removal operations, representing a significant biomass loss in addition to that caused by natural predatory activity. Thus the results of this study could represent an opportunity to replenish the heavily depleted natural stock. Additionally, the ability to raise embryos in captivity from these recovery efforts offers a tangible opportunity for new economic investments in addition to being an action with a significant ecological impact. Indeed, data coming from one small fishery in the north-western coast of the Adriatic Sea, estimated that the loss caused by egg laying on traps reached 3.5 million eggs [18]. Since several issues should be solved before the breeding of this species in captivity can easily occur, observing the eggs laid by females on traps or ropes and brought ashore by fishermen could be the first step for stemming the observed decline of natural stocks. Thus, the present study attempted to identify a dry diet, among those commercially available, to be administered to cuttlefish hatchlings during early phases of development as a starting point to encourage a type of more sustainable, extensive aquaculture, developed according to the recovery of natural resources. Nevertheless, the proposed diet is much cheaper than the fresh one, and, above all, the preparation and administration time is lower.

## 2. Materials and Methods

### 2.1. Cuttlefish Collection and Rearing

Clusters of cuttlefish (*Sepia officinalis*) eggs, laid on trap or ropes were collected during spawning season (April–May 2022) in two different fishing sites close to Ancona, Marche Region, Central Italy (43°33′52″ N 13°35′28″ E and 43°35′39″ N 13°30′12″ E), and transferred to the university’s facility (DiSVA, UNIVPM) in marine water in a polystyrene box to maintain temperature. In order to collect a sufficient number of hatchlings [14], up to 25 egg clusters, each made up of at least 100 eggs, were maintained in 6 rectangular tanks (0.41 m^2^, 75 × 55 cm). The water column was 16 cm high. Tanks were equipped with an aeration system and mechanical and biological filters. Total water filtration occurred every 3 h and water parameters were set as follows: salinity 40 PSU, temperature 20 °C, photoperiod 13:11 L/D (300 lux) [19], pH 8, the absence of ammonia and nitrites, and nitrates <20 mg/L. Rearing conditions were selected considering the recent advances in cuttlefish culture [20]. Egg clusters were left attached to the ropes until they naturally hatched.

### 2.2. Experimental Diets and Sampling

All the eggs that hatched within the 6 tanks over the course of 3 h were pooled. Three different collections were made in order to have 3 different batches of hatchlings. Hatchings from each collection (N = 300), were divided among 10 tanks, each containing 30 cuttlefish (77 ind./m^2^ stocking density), and 5 different diets, in duplicates, were administered, based on their nutritional requirements [21], as follows: (1) Diet A—frozen mysis (Eschematteo, Parma, Italy), (2) Diet B—commercial feed containing 6% krill (Natura 3/5, Inve Technologies, Dendermonde, Belgium), (3) Diet C—commercial feed containing 20% krill (500 µm pellet) (Fish Breed-M, Inve Technologies, Dendermonde, Belgium), (4) Diet D—100% dried krill (Tetra, Melle, Germany), and (5) Diet E—dried gammarids (Naturalpet, Melicucco, RC, Italy). Details on diet nutritional values, as reported by the producers, are shown in Supplementary Table S1. Cuttlefish were fed ad libitum 3 times per day during the 10-day trial and the feeding ratio was not less than 10% of the fresh weight. During this experimental phase, mortality was monitored daily.

For all the analyses below described (morphological parameters, histology, enzymatic activity, and molecular biology assays), 5 cuttlefish/analysis per collection were sampled immediately after hatching (T0). At 5 (T1) and 10 (T2) dph, 5 cuttlefish were randomly collected from duplicate tanks/collection (N = 15 per experimental diet/sampling time) for each of the analyses described. A graphical representation of the experimental design is shown in Supplementary Figure S1.

All procedures involving animals were conducted in accordance with the Italian law on animal experimentation and were approved by the Ethics Committee of the Universita’ Politecnica delle Marche, Ancona, Italy, and by the Italian Ministry of Health (Aut. No. 488/2020-PR). Cuttlefish were sacrificed using an overdose of anesthetic (MS-222, 1 g/L). All efforts were made to minimize animal suffering and the study was carried out in compliance with the ARRIVE guidelines.

### 2.3. Morphological Studies

Morphometric data (total length—TL, including arms and dorsal mantle length—DML) were collected using a stereomicroscope (Leica Wild M3B, Meica Microsystem, Buccinasco, Milan, Italy). A Deltapix camera and its software (Deltapix InSight V6.5.2, Smorum, Denmark) were used to record the image [22]. Total dry weight—DW—was obtained by specimen lyophilization using an LIO-5P apparatus (CinquePascal srl, Trezzano sul Naviglio, Milan, Italy) and recorded using a digital balance (Mettler-Toledo, Milan, Italy).

### 2.4. Cuttlefish Gut Histology

Histological analyses were performed according to [23]. Briefly, cuttlefish (n = 5 for each experimental tank) were fixed in Bouin’s solution (Sigma Aldrich, MO, USA) and stored at 4 °C for 24 h. Samples were then dehydrated by graded ethanol solutions, washed with xylene (Bio-Optica, Milan, Italy), and embedded in solid paraffin (Bio-Optica, Milan, Italy). Moreover, 5 μm sections were obtained by a rotary microtome (Leica RM2125 RTS, GmbH, Wetzlar, Germany) and stained using Mayer hematoxylin and eosin Y (H&E) (Sigma Aldrich, MO, USA). Slides were examined under a Zeiss Axio Imager. We used an A2 microscope (Zeiss, Oberkochen, Germany) combined with a color digital camera Axiocam 503 (Zeiss, Oberkochen, Germany). Histo-morphometric parameters such as fold height and width, as well as cilia length, were measured.

### 2.5. Digestive Enzyme Activity

Amylase and alkaline phosphatase activity were assayed as previously described [24]. Lipase activity was assayed as described in [25] according to a modified method [26] using β-naphtyl caprilate as substrate. Since the cuttlefish small size and the impossibility of exciding guts, whole embryo extracts were used for the assay. Enzyme activities were normalized to the protein content of homogenates, which was determined according to [27] using bovine albumin serum as a standard.

### 2.6. RNA Extraction, cDNA Synthesis and Real Time PCR Analysis

For each experimental group, total RNA was extracted from 5 different hatchlings (N = 5) using RNAzol (Merck, Darmstad, Germany) as previously described in [28]. Briefly, each hatchling was homogenized using an Ultra-Turrax homogenizer (IKA-Werke GmbH & Co., Staufen, Germany), total RNA was extracted according to the standard protocol, and genomic DNA contamination was removed with DNase I digestion. RNA was then run on 1% agarose gel to verify its quality and quantified using a nanodrop spectrophotometer (NanoPhotometer™ P-Class, IMPLEN, München, Germany). Moreover, 5 µg of total RNA was used for cDNA synthesis, using Iscript cDNA synthesis kit (Biorad, Milan, Italy).

The qRT-PCRs were performed with the SYBR green method in a CFX thermal cycler (Bio-rad, Milan, Italy) as previously described in [29]. For each experimental group, replicates (N = 5) were run in duplicate. The final primer concentration was 10 pmol/μL. β tubulin (βtub) mRNA and 18S RNA were selected using NormFinder (V0.953) [30] and used to normalize target gene expression levels analyzed by CFX Manager Software version 3.1 (Bio-Rad, Hercules, CA, USA), including GeneEx Macro Conversion and GenEx Macro files, and the results are represented by bar plots along with the standard deviation. The primer sequence is shown in Table 1.

### 2.7. Statistical Analysis

All statistical analyses were performed using the statistical software package Graph Pad Prism V9.0.1. (GraphPad Software, Inc., San Diego, CA, USA) with significance accepted at *p* < 0.05. A two-way ANOVA non-parametric test, followed by the Tukey test as a multiple comparisons test, was used to compare changes among the groups. Letters/asterisks indicate statistically significant differences among experimental groups.

## 3. Results

### 3.1. Feeding Trials

**Figure 1:**
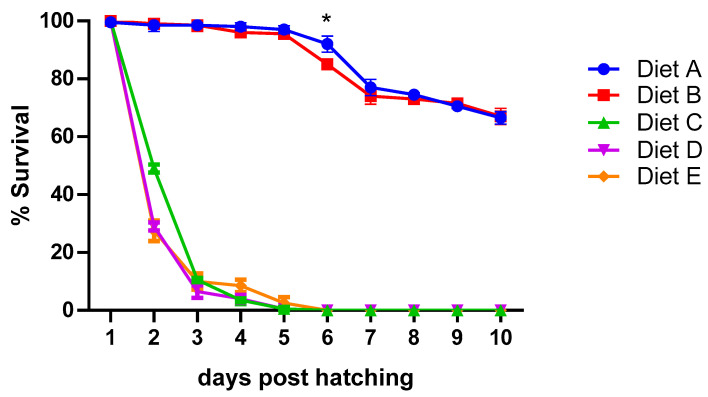
Daily survival in cuttlefish fed the five different diets. Survival is shown as mean ± SD of each experimental tank (two tanks for each of the three hatchling collections) and is expressed as % of cuttlefish died/total cuttlefish (N = 6). An asterisk * denotes statistically significant difference (*p* < 005) between cuttlefish fed Diets A and B.

As shown in Figure 1, high mortality was registered in tanks fed Diets C, D, and E starting from day 2, reaching the 100% at day 5 (T1). Differently, a similar survival rate (≥99%) was observed for both Diets A and B till 3 dph. Starting from 4 dph, in cuttlefish receiving Diet B, a lower survival rate was observed, which was significantly lower only at 6 dph. At day 6, survival was higher than 80% for the Diet B group and around 95% in the Diet A-fed cuttlefish. Starting from 8 dph, survival decreased similarly in both experimental groups, resulting in the same value (>60%) at T2. Given that all cuttlefish fed diet C, D, and E died within day 5, statistical analysis was performed only comparing the survival rate of Diets A and B.

**Figure 2:**
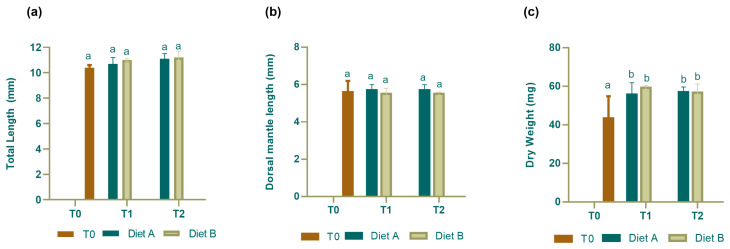
Morphometric parameters. (**a**) Total Length (TL) and (**b**) Dorsal Mantle Length (DML) and (**c**) dry weight in *S. officinalis* at hatching (T0) and fed Diet A and Diet B at T1 (5 dph) and T2 (10 dph). Data are expressed as mean ± SD (N = 15). Different letters indicate statistically significant difference (*p* < 005) among all groups.

During the 10-day trial, for both sampling time, no significant changes in TL (Figure 2a) and DML (Figure 2b) were observed in groups fed the two different diets with respect to hatching time (T0). Regarding dry weight, the administration of both diets caused a similar weight gain at either T1 and T2, with respect to T0 (Figure 2c).

### 3.2. Histological Results

**Figure 3:**
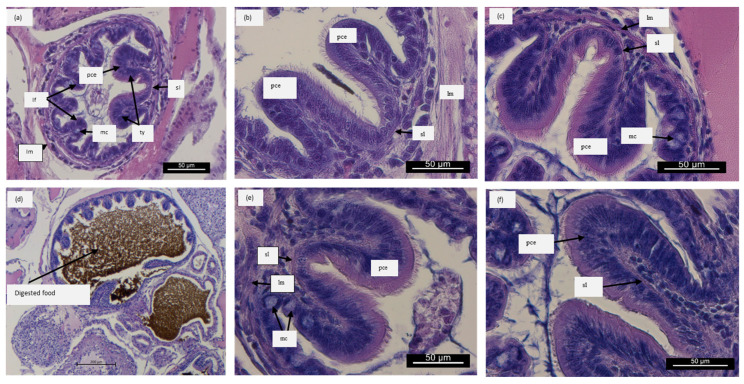
(**a**) Panoramic view of the intestine showing circular longitudinal muscle (lm), typhlosoles (ty), and longitudinal folds (lf) extending to the cavity at T0. Mucous cells (mc), pseudostratified ciliated epithelium (pce), submucosal layer (sl). (**d**) Panoramic view of an intestine section showing the presence of digested food. Scale bar: 200 μm. Representative light microscopic images depicting morphological changes in intestine of cuttlefish fed Diet A at 5 (T1) (**b**) and 10 (T2) (**e**) dph and in those fed Diet B at 5 (**c**) and 10 (**f**) dph. Scale bar: 50 μm.

Figure 3a shows a panoramic view of cuttlefish intestines with typical morphological structures and architectures. Figure 3d shows the presence of digested food within the intestine to confirm that cuttlefish were fed the two artificial diets. The video clearly shows cuttlefish eating the dry pellets (Supplementary Video S1) The arrows point out the main morphological structures in organisms receiving Diet A (Figure 3b, T1, and Figure 3e, T2) and in organisms receiving Diet B (Figure 3c, T1, and Figure 3f, T2).

The main caecum structures are measured and the data are reported in Table 2. Concerning fold size, a similar increase in height, with respect to T0, was observed, regardless the diet, at 5 dph (T1). However, at 10 dph (T2), only in specimens fed Diet B was a significant increase, with respect to Diet A, observed. At T2, cuttlefish fed Diet A did not show differences with respect to T1. Fold width significantly increased only in cuttlefish fed Diet B at 10 dph (T2) with respect to T0 and T1. Cilia height was not affected by the two diets at each of the time points analyzed.

### 3.3. Digestive Enzyme Activity

**Figure 4:**
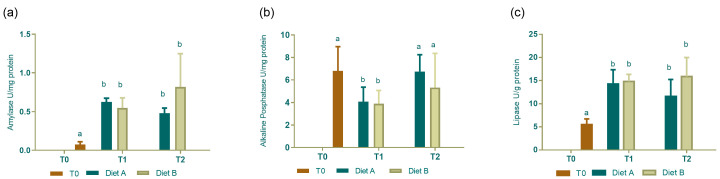
Digestive enzyme activity. (**a**) Amylase, (**b**) alkaline phosphatase, and (**c**) lipase levels in cuttlefish at hatching (T0) and fed Diet A and Diet B at 5 (T1) and 10 dph (T2). Data are reported as mean ± S.D; N = 15. Different letters indicate statistically significant difference (*p* < 005) among all groups.

Amylase activity rose from T0 to T1 and T2. No differences in the levels were measured between the two diets neither at 5 nor at 10 dph (Figure 4a). Differently, alkaline phosphatase levels decreased from T0 to T1, and T2 presents similar value to hatching. No difference were measured between the two feeding diets at each of the two time points analyzed (Figure 4b). Regarding lipase, its levels increased from hatching to T1 and T2. No statistically significant differences were detected between the two diet enzyme levels at T1 and T2, although activity seemed to decrease in cuttlefish fed Diet A and increase in those fed Diet B at T2, with respect to T1 (Figure 4c).

### 3.4. Real-Time PCR of Genes Involved in Stress Response and Antioxidant System

**Figure 5:**
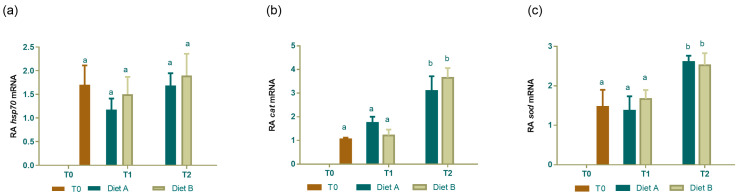
Expression profiles of stress and oxidative stress response biomarkers. (**a**) hsp70, (**b**) cat, (**c**) sod mRNA in cuttlefish at hatching (T0) and fed Diet A and Diet B at 5 (T1) and 10 dph (T2). mRNA levels were normalized against 18S and β-tubulin used as reference genes. Data are shown as the mean ± SD. Different letters indicate statistically significant differences among the experimental groups (*p* < 0.05). RA: relative abundance.

By real-time PCR, the expression of genes involved in stress response as well as in the antioxidant system activation revealed that the two different diets did not affect the expression of selected mRNAs. In detail, *hsp70* (Figure 5a) mRNA levels remained stable between hatching and 5 and 10 dph and did not change between the two diets. Considering the antioxidant genes, both cat (Figure 5b) and sod (Figure 5c) transcript levels presented similar levels between T0 and T1 and then significantly increased from T1 to T2, but no significant differences in transcript expression were observed between the two different diets administered at either of the two time points fixed.

## 4. Discussion

The results herein described show preliminary evidence regarding the successful feeding of hatchlings with a dry pelleted diet, providing encouraging indications for the possibility of undertaking the large-scale breeding of this species, whose market demand is increasing, thus causing a significant reduction in wild stock. Noteworthy, this diet guarantees the same performance as a diet based on mysis (Diet A), which represents our positive control, as it is qualitatively closer, from a nutritional standpoint, to what the cuttlefish feed on in their natural environment but offers multiple advantages starting from its commercial availability, lower costs, and the ease of administration by all operators. On the contrary, the high mortality observed in cuttlefish fed the other diets could be caused by an immediate depletion of the vitellogenin reserves or by a possible adverse effect on their digestive system caused by these diets. Noteworthy is the 6% of krill content in diet B, which represents a food source with high nutritional value relevant for both cuttlefish and human diets. Krill indeed is a rich source of high-quality protein, with the advantage over other animal proteins of being low in fat and rich in omega-3 fatty acids [31]. In addition, krill presents higher levels of antioxidants [32], which could limit the oxidative damage occurring during development and growth. Differently from the other commercial diets used in the preliminary feeding trial, Diet B contains higher levels of P and some trace elements, mainly Fe, Mn, and Zn. Trace elements are known to be essential elements whose role in the etiology and prevention of chronic diseases has been well described [33], while P is essential for growth as part of a wide variety of organic phosphates such as nucleotides (particularly ATP), phospholipids, and coenzyme. Therefore, the selection of dietary sources of P has received special attention for fed formulation in aquaculture [34]. Recently, Lemos and collaborators observed higher apparent digestibility coefficients and higher growth in shrimp fed with monocalcium phosphate and monoammonium phosphate [35], suggesting the beneficial role of P also on cuttlefish growth. Moreover, recent studies conducted in sea urchin and Pacific oyster, although being two species that significantly differ from cuttlefish, revealed that a correct synergy between the different trace elements is of paramount importance for correct larval development [36,37], thus having the potential to positively boost growth also in cuttlefish hatchlings. Nevertheless, despite the fact that Diets B and C present a similar composition, they have a different DHA/EPA ratio, which is higher in Diet B. While no data are currently available regarding the role of DHA/EPA in cuttlefish nutrition, several studies so far have demonstrated that in teleost, a ratio >2 guarantees a higher survival rate [38,39]. The analysis of molecular markers provided an overall scenario regarding the health status of the reared larvae and pinpointed a similar condition between the two diets, underlying that Diet B could successfully replace Diet A and did not create the onset of stress to organisms. Variation in the levels of *hsp70* mRNA is indeed a clear signal of the presence of stressful events [40], from incorrect breeding and rearing conditions [41] to exposure and environmental pollutants [42]. In addition, the transition from the embryonic to juvenile stage is accompanied by differences in metabolic, locomotor, and feeding activities that can reflect the fish’s oxidative status. The modification of digestive mechanisms and transition to novel food sources together with intense body growth and development are processes responsible for changes in metabolic activity and consequently reactive oxygen species (ROS) production [43]. In this contest, the results herein obtained suggest that both diets similarly modulate the expression of antioxidant genes. As suggested by histological analysis and enzymatic activity assays, both diets guarantee similar gut development. Important is the detection of amylase, a key enzyme for the digestion of complex carbohydrates and glycogen, the activity of which is generally high at the first feeding and tends to decrease as the development progresses [44], resulting as a valuable indicator of the fish’s digestive capacity and its nutritional and physiological state [44]. From a practical point of view, the ability of fish to produce amylase is a great benefit for aquaculture research and the industry, which use starch as a cheap energy source and as a binder in formulated diets [45] and, in our case, had involvement in the maturity of the cuttlefish’s gut. At the same time, the similar levels of alkaline phosphatase, an evolutionarily conserved system with a pivotal role in controlling gut and systemic inflammation, exclude the possibility that the pellet diet could have an inflammatory effect on gut. In production animals, inflammation is detrimental to energy balance and represents a metabolic cost that can significantly limit production efficiency [46]. Finally, the trend observed for lipase activity supports the increase in the use of lipids as the development proceeds, as suggested by previous studies showing the important role of fatty acids in cephalopod hatchling nutrition [47,48]. We can speculate that at 5 dph, most of the lipids necessary for growth derive from yolk, which is still pretty abundant, while possibly at 10 days, cuttlefish start using lipids supplied with the diet, thus explaining the rise observed.

## 5. Conclusions

In conclusion, our results provide some preliminary evidence regarding the possibility of rearing cuttlefish in captivity using a pelleted diet. This could be the starting point to motivate aquacultures toward this species rearing, providing a farmed product as a valid alternative to wild catches or for restocking practices. Although this is very promising, further research is still needed to assess the effect of diets later in development, the possibility of reaching sexual maturity, and, no less important, the possibility of testing the taste of the product, which are very important variables for successful commercial aquaculture production.

## Figures and Tables

**Table 1:** Primer list.

mRNA	Primer Sequence	Annealing T
*βtub for*	5′ GGTGCTTGTCAAGGTCCCTA 3′	56
*βtub rev*	5′ GTCAACAGCAACCAGTTTCCT 3′	
*18S for*	5′ GTCGGTTTTCTCACGCACTT 3′	57
*18S rev*	5′ CGGGAGGTGGTTAAGAGGTT 3′	
*cat for*	5′ TTCGTTTCTCTACCGTTGGTG 3′	58
*cat rev*	5′ AAGTCCCAGTTACCGTCTTCC 3′	
*Cu-Zn sod for*	5′ GAGACTTTCGTTAGGACGGATA 3′	56
*Cu-Zn sod rev*	5′ AGCCATTCCCCTTATTTCAC 3′	
*hsp70 for*	5′ GAGTTCAAGCGAAAGCACA 3′	59
*hsp70 rev*	5′ AGCGACCTACAAGGACAAT 3′	

**Table 2:** Caecum morphological parameters in *S. officinalis* fed Diet A and B. Data are presented as mean ± S.D; (N = 15). Different superscript letters in each row denote statistically significant differences among all groups (*p* < 0.05).

	Treatments				
		Diet A		Diet B	
Analyzed Parameters	T0	T1	T2	T1	T2
Fold height (μm)	100.6 ± 2.0 ^a^	124.2 ± 7.8 ^bc^	122.2 ± 4.8 ^c^	122.6 ± 4.3 ^c^	140.0 ± 8.9 ^b^
Fold width (μm)	61.0 ± 1.1 ^a^	66.5 ± 2.9 ^ab^	64.0 ± 1.1 ^a^	62.5 ± 1.8 ^a^	70.9 ± 2.6 ^b^
Cilia height (μm)	6.0 ± 0.2 ^a^	5.9 ± 0.3 ^a^	6.2 ± 0.2 ^a^	5.7 ± 0.3 ^a^	5.9 ± 0.3 ^a^

## Data Availability

The data presented in this study are available on request from the corresponding author. The data are not publicly available due to privacy reasons.

## Supplementary Materials

The following supporting information can be downloaded at: https://www.mdpi.com/article/10.3390/ani14131993/s1, Figure S1: Representation of the experimental design; Table S1: Composition of commercially available diets as reported by producers; Video S1: Hatchlings hunting for dry Food.
