# Supplementary material for: First Feeding of Cuttlefish Hatchlings: Pioneering Attempts in Captive Breeding
Source: Animals (Basel). 2024 Jul 6;14(13):1993. doi: 10.3390/ani14131993 (PMC11240666; doi:10.3390/ani14131993)
Supplement: Supplementary file 1 [file animals-14-01993-s001.zip › animals-3037744-SI.zip!/animals-3037744-Supplementary Materials.pdf]

Supplementary Materials

	Diet B	Diet C	Diet D	Diet E
**Size**	300-500 µm	powder	0.5-1 cm	1 cm
**Krill %**	6	2	100	
**Gammarus %**	0	0	0	100
**Crude Protein**	50	59	62	41
**Crude oils and fats**	14	15	4	3
**Humidity**	/	/	6	9
**Crude cellulose**	/	/	4	8
**Crude ash**	12	13		25
**Ash insoluble in HCl**	2	4.2		
**Ca**	1	1.7		
**P**	196	1.5		
**Na**	0.9	0.9		
**Crude fibre (mg/g dwt)**	0.5	1		
**DHA**	22.5	22		
**EPA**	7.5	10		
**Additive Vitamins IU/kg**				
Vit. A (3a672b)	5000	25000		
Vit. D3 (3a671)	10	5000		
**Additive Vitamins mg/kg**				
Vit. E	700	600		
Vit. C	500	5000		
**Additive Trace Elements mg/kg**				
Fe (ferric oxide) E1	560	0		
I (calcium iodate, anhydrous, E2)	5	5		
Cu (Cu chelate of glycine, hydrate, E4)	6	5		
Mn (Mn chelate of glycine, hydrate, E5)	50	20		
Zn (Zn chelate of glycine, hydrate, E6)	50	30		
Se (selenomethionine produced by Saccharomyces cerevisiaeNCYC R646, 3b8.13)	0.3	0.3		
**Additive Antioxidants mg/kg**				
Ethoxyquin (E324)	100	120		
BHA (E320)	25	20		
Propyl gallate (E310)	25	10		
**Additive Colourants mg/kg**				
Astaxanthin (2a161j)	0	20		

Supplementary Table S1 Composition of commercially available diets as reported by producers. Diet B (Natura 3/5, Inve technologies, Dendermonde, Belgium), Diet C (Fish breed-M, Inve technologies, Dendermonde, Belgium), 4) Diet D (Tetra, Melle, Germany), 5) Diet E (Naturalpet, Melicucco, RC, Italy).

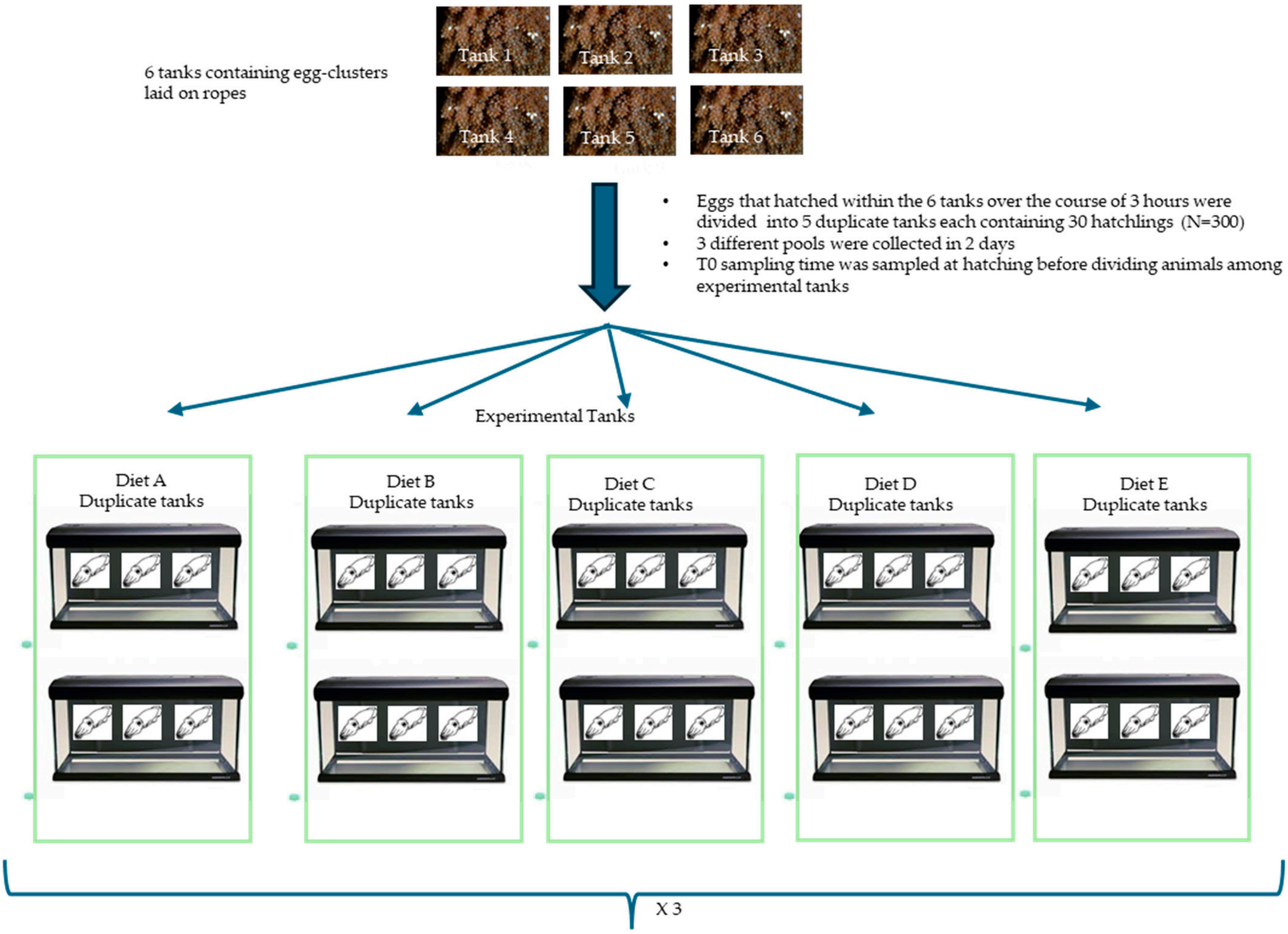

5 cuttlefish were randomly sampled from the two duplicate tanks set up for each experimental diet T1 and T2, for each of the analyses described in M&M

In details, 5 cuttlefish for morphometric analysis,
- ✓ 5 for histology,
- ✓ 5 for enzymatic activity assay
- ✓ 5 for molecular biology

Supplementary Figure S1 Representation of the experimental design
